# Bilateral Differential Topography—A Novel Topographic Algorithm for Keratoconus and Ectatic Disease Screening

**DOI:** 10.3389/fbioe.2021.772982

**Published:** 2021-12-09

**Authors:** Yang Shen, Yiyong Xian, Tian Han, Xuanqi Wang, Xingtao Zhou

**Affiliations:** ^1^ Eye Institute and Department of Ophthalmology, Eye and ENT Hospital, Fudan University, Shanghai, China; ^2^ NHC Key Laboratory of Myopia, Fudan University, Shanghai, China; ^3^ Key Laboratory of Myopia, Chinese Academy of Medical Sciences, Shanghai, China; ^4^ Shanghai Research Center of Ophthalmology and Optometry, Shanghai, China; ^5^ Shanghai Engineering Research Center of Laser and Autostereoscopic 3D for Vision Care (20DZ2255000), Shanghai, China

**Keywords:** corneal ectasia, forme fruste keratoconus, keratoconus, pachymetry, topography

## Abstract

**Purpose:** The purpose of this study was to establish a novel bilateral differential topographic algorithm and assess its efficacy for screening of keratoconus and corneal ectasia before corneal refractive surgery.

**Methods:** One hundred and sixty-one consecutive patients (115 men and 46 women, aged 22.8 ± 6.8 years) with keratoconus, including clinical keratoconus, subclinical keratoconus, forme fruste keratoconus (FFK), and corneal ectasia (KC group) and one hundred and seventy-four consecutive patients (97 men and 77 women, aged 25.1 ± 6.7 years) with ametropia (control group) visiting the Eye and ENT hospital of Fudan University from June 2018 to April 2021 were included. Bilateral differential keratometry, elevation, and pachymetry topographies were composed based on raw topographic data obtained by a Scheimpflug imaging anterior segment analyzer. Key bilateral differential characteristic parameters were calculated. SPSS 20 (SPSS Inc., IBM) was used for statistical analyses and the receiver operating characteristic (ROC) curves were used to determine the diagnostic efficacies.

**Results:** Mann-Whitney tests detected that the front keratometry, front elevation, corneal pachymetry, and back elevation maximal, mean, and standard deviation values within a 1.5-mm radius of the bilateral differential topography were all significantly higher in the KC group than in the control group (all *p*-values <0.001). The front keratometry mean (ΔFKmean) and standard deviation (ΔFKsd) and the front elevation standard deviation (ΔFEsd) and maximal (ΔFEmax) values within a 1.5-mm radius of the bilateral differential topography yielded the four highest accuracies (area under the ROC curve = 0.985, 0.985, 0.984, and 0.983, respectively) for discriminating KC cases (including FFK cases) from normal cases. Cut-off values of 0.75 diopters (D) for the ΔFKmean, 0.67 D for the ΔFKsd, 2.9 μm for the ΔFEsd, and 14.6 μm for the ΔFEmax had the highest sensitivities (95.7, 95.0, 96.9, and 95.0%, respectively) and specificities (96.0, 97.7, 94.8, and 95.4%, respectively).

**Conclusion:** Bilateral differential topographic parameters may be efficient for the early detection of keratoconus and corneal ectasia secondary to corneal refractive surgery. This bilateral differential topographic algorithm may complement conventional diagnostic models by improving the sensitivity and specificity of screening for early keratoconus and ectasia before corneal refractive surgeries.

## Introduction

Keratoconus and ectatic diseases are characterized by an abnormal thinning and conic protrusion of the cornea that could result in significant visual impairment. Common risk factors for keratoconus include family history, ocular allergy, constant eye rubbing, as well as connective tissue disorders. These diseases often go undetected initially, and they tend to progress from one eye to the other, with a prevalence of approximately 1/2000 in the general population (Gomes et al., 2015a; Gomes et al., 2015b; Naderan et al., 2017). Ruptures in the stromal lamella and breakage of collagen fibers are believed to be the main underlying mechanisms for the compromised corneal biomechanical stability observed in these diseases (Krachmer et al., 1984; Alkanaan et al., 2019).

Corneal topographic and tomographic assessments are useful for the diagnosis of keratoconus and ectatic diseases (Buhren, 2014; Yip and Chan, 2019). Initially, Amsler introduced the Amsler–Krumeich diagnostic and classification system to assess the severity of keratoconus based on the mean corneal power, astigmatism, transparency, and corneal thickness (Amsler, 1946). Though this system improved the accuracy of diagnosing keratoconus, demonstrating a sensitivity of 89.3% and specificity of 71.9% (Gobbe and Guillon, 2005; Saad and Gatinel, 2016), its accuracy was still too low for screening keratoconus prior to corneal refractive surgery.

A Scheimpflug imaging-based anterior segment analyzer is another commonly used tool for screening keratoconus and corneal ectasia in recent decades, especially prior to corneal refractive surgery. With more than 100,000 true elevation points collected from the anterior surface of the cornea to the posterior surface of the crystalline lens, this analyzer provides various topographic parameters, including the anterior keratometry, posterior keratometry, corneal pachymetry, and anterior and posterior corneal elevations (Chan et al., 2021). Based on these topographic parameters, Belin MW introduced the ABCD diagnostic and classification system (Belin and Duncan, 2016) and the Belin/Ambrósio Enhanced Ectasia total deviation index (BAD), which further improved the accuracy of identifying a susceptibility to corneal ectasia (with an 87.3–98.9% sensitivity and a 97.5–99.8% specificity). However, the diagnostic sensitivity and specificity for identifying forme fruste keratoconus (FFK), a topographically normal fellow eye with unilateral clinical keratoconus, was significantly decreased to 89.2 and 81.3%, respectively, with these tools (Muftuoglu et al., 2015; Lopes et al., 2018).

Anterior segment optical coherence tomography (AS-OCT) is another noninvasive, three-dimensional imaging technique that yields a higher resolution (up to 1 µm) than Scheimpflug tomography. Previous studies have reported that OCT-generated corneal and epithelial thickness maps demonstrate outstanding sensitivities (higher than 97%) and specificities (100%) for diagnosing keratoconus. However, the sensitivity for FFK screening is significantly lower, at 73.7% although the specificity remains 100% (Yang et al., 2021). Recently, big data and artificial intelligence algorithms (including deep learning and machine learning) provided new approaches to diagnose keratoconus and corneal ectasia through the recognition of typical tomographic and topographic features (Arbelaez et al., 2012; Abdelmotaal et al., 2020). However, studies with large sample size have shown that the diagnostic accuracy of these methods for identifying early keratoconus, including FFK and at-risk corneas, is unsatisfactory (Ruiz Hidalgo et al., 2016).

Until now, although all the above-mentioned mainstream models have diagnosed keratoconus and corneal ectasia unilaterally, the evaluation of bilateral asymmetry in corneal topography and tomography has been neglected. However, intereye asymmetry in the corneal shape is a key characteristic of keratoconus, especially FFK (in which one eye is clinically keratoconic, but the other eye is topographically normal), which relies heavily on a comparison of bilateral topographies (Burns et al., 2004; Li et al., 2004; Galletti et al., 2015). The present study aimed to establish a novel bilateral differential topographic algorithm using raw data related to elevation, keratometry, and pachymetry obtained using a Scheimpflug imaging device. Furthermore, we investigated the clinical value of this new algorithm for the diagnosis of keratoconus and corneal ectasia.

## Materials and Methods

### Participants and Materials

In this case-control study, 161 consecutive patients (115 men and 46 women) with keratoconus (including clinical keratoconus, subclinical keratoconus, and FFK) or corneal ectasia secondary to corneal refractive surgery (KC group) were compared with 174 (97 men and 77 women) consecutive patients with ametropia (control group). The mean ages of the KC and control groups were 22.8 ± 6.8 years and 25.1 ± 6.7 years, respectively. Keratoconus was diagnosed according to the Global Consensus on Keratoconus Diagnosis from 2015 (Gomes et al., 2015a) by an experienced ophthalmologist, XTZ. The raw topographic data for all cases were exported from a Scheimpflug-based anterior segment analyzer (Scansys, Mediworks, Shanghai, China) at the Eye and ENT Hospital of Fudan University from June 2018 to April 2021.

### Topographic Examination

The anterior segment analyzer used in this study was equipped with one Scheimpflug camera. By rotating 180° in less than 3 s, the camera was capable of capturing more than 60 tomographic images with 230,400 data points to form a front keratometry matrix (FKM), back keratometry matrix (BKM), front elevation matrix (FEM), back elevation matrix (BEM), corneal pachymetry matrix (CPM), iris matrix, and crystalline lens matrix, thus reconstructing a three-dimensional anterior segment model.

For this study, patients were asked to focus on a target (a blue light) in the analyzer, with the chin on a chin rest and the forehead against a forehead strap. The measurement interface guided the operator to aim at the vertex of the patient’s cornea with a joystick attached to the analyzer, and a Scheimpflug camera automatically rotated and captured tomographic images of the anterior segment. Key parameters, including the front flat keratometry (FK1), front steep keratometry (FK2), front mean keratometry (FKm), front maximum keratometry (FKmax), back flat keratometry (BK1), back steep keratometry (BK2), back mean keratometry (BKm), back maximum keratometry (BKmax), corneal thickness at the thinnest point (TCT), and corneal thickness at the vertex (CTV), were also calculated and recorded.

### Algorithms for Bilateral Differential Topography and Bilateral Differential Characteristic Parameters

**FIGURE 1:**
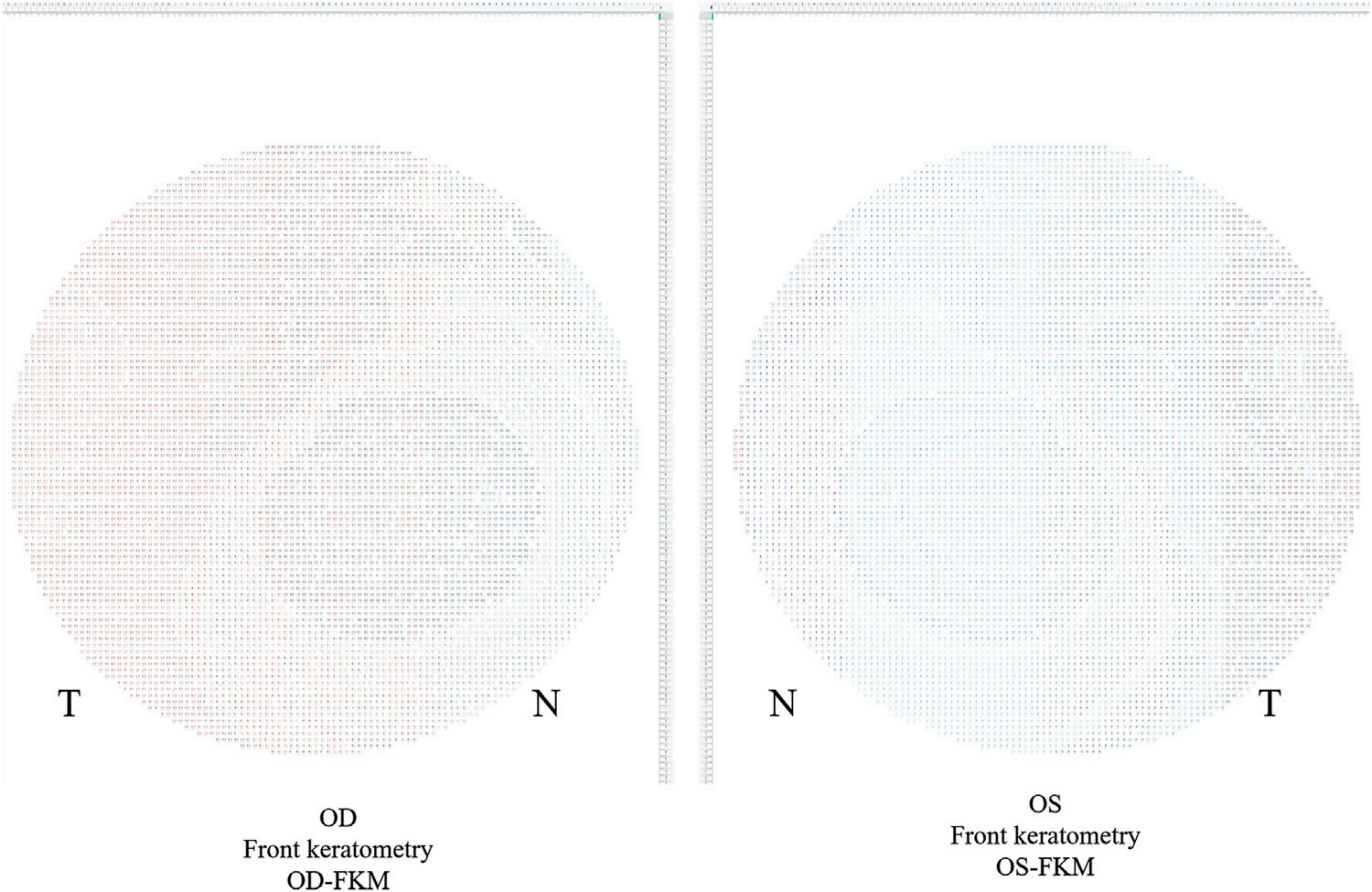
Exported binocular front keratometry matrix (FKM) data.

**FIGURE 2:**
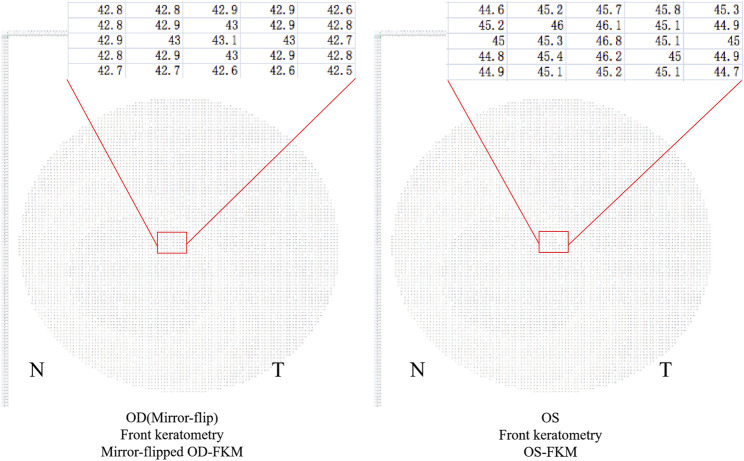
Mirror-flipped OD-FKM established for calculation. The red box shows a part of raw data in the FKM.

**FIGURE 3:**
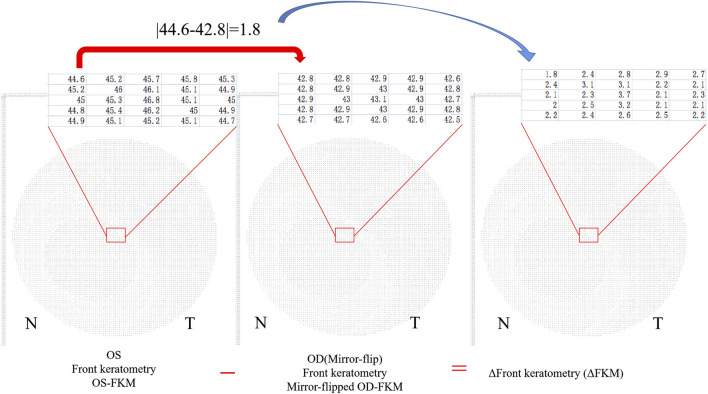
Bilateral differential FKM (ΔFKM) obtained by calculating the absolute value of the OS-FKM minus the mirror-flipped OD-FKM.

**FIGURE 4:**
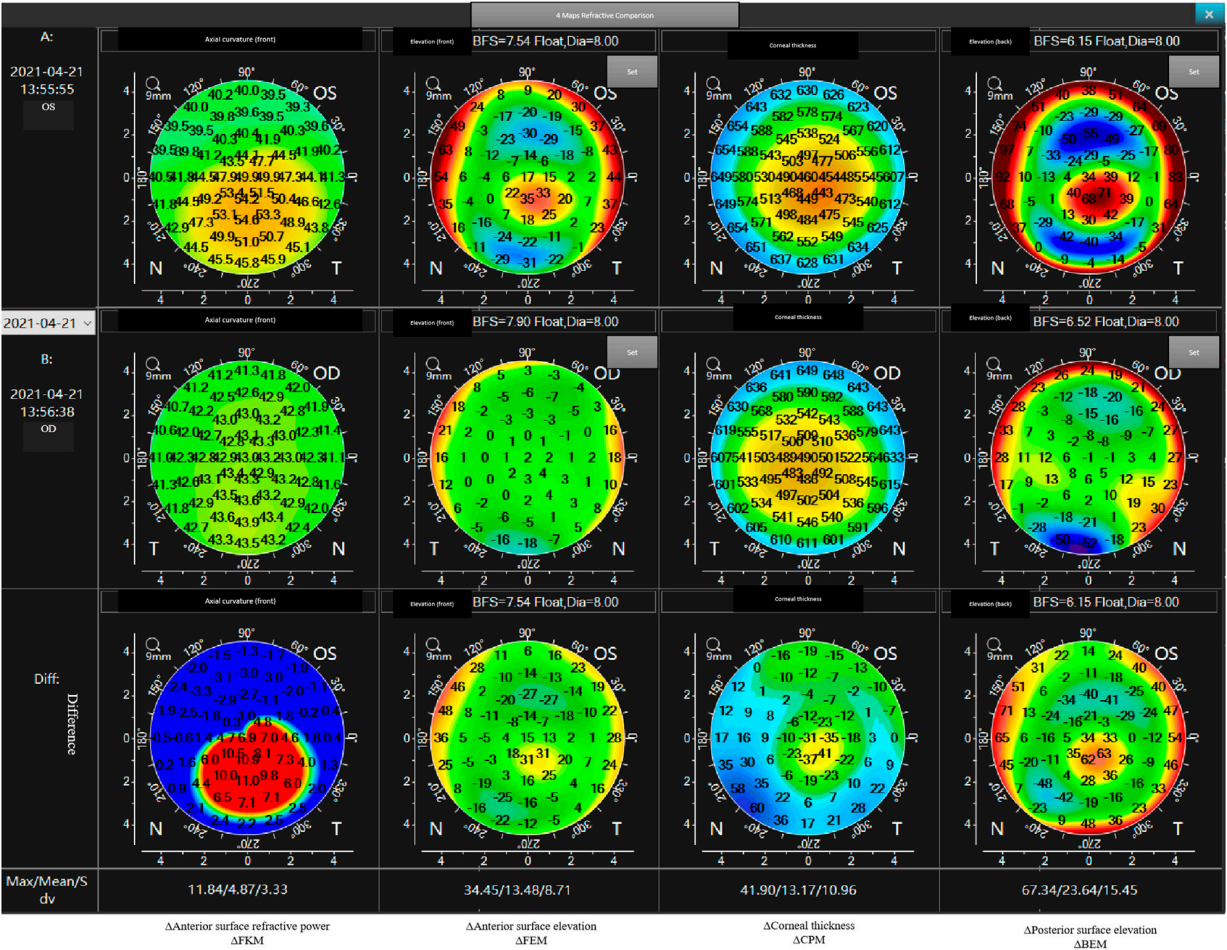
An example of bilateral differential topographic display of a patient with keratoconus.

The method for analyzing the bilateral differential topography of the FKM is presented next to illustrate the algorithms used in this study:

1) Export binocular FKM (OD-FKM and OS-FKM) data for each case to EXCEL files (Microsoft), with each matrix containing 121 × 121 cells filled with front keratometry values (Figure 1).
2) Freeze the central cell, which is located at Row 61/Column 61 (61, 61), for each matrix in the EXCEL files. The space between cells is 0.1 mm.
3) Mirror flip the OD-FKM to obtain a mirror-flipped OD-FKM (Figure 2).
4) Determine the bilateral differential FKM (ΔFKM) by calculating the absolute value of the OS-FKM minus the mirror-flipped OD-FKM (Figure 3). ΔFEM, ΔBEM, and ΔCPM were also determined using the same algorithm mentioned above. Figure 4 shows an example of bilateral differential topographic display of a patient with keratoconus.
5) Abstract all cells from the central cell (61, 61) to the cells located within a 1.5-mm radius of the ΔFKM, thus composing a matrix of ΔFKM SUB.
6) Acquire bilateral differential characteristic values, including the ΔFKmax (which refers to the maximum value of ΔFKM SUB), ΔFKmean (which refers to the mean value of all the cells in the ΔFKM SUB, or ΔFKmean = $\overset{k=N}{\underset{k=1}{\sum }}\left({\text{ΔFKM SUB}}_{k}\right)/N$ ; N = the number of cells), and the ΔFKsd (which refers to the standard deviation of all the cells in the ΔFKM SUB, ΔFKsd = $\sqrt{\frac{1}{N}\overset{k=N}{\underset{\text{k}=1}{\sum }}{\left({\text{ΔFKM SUB}}_{k}-\text{ΔFKmean}\right)}^{2}}$ , N = the number of cells). ΔFEmax, ΔFEmean, ΔFEsd, ΔBEmax, ΔBEmean, ΔBEsd, ΔCPmax, ΔCPmean, and ΔCPsd were also calculated using the same formulas.

### Statistical Evaluation

SPSS 20 (SPSS Inc., IBM) was used to perform statistical analyses. Normality checks were conducted using the Kolmogorov-Smirnov *Z* test. The differences in the mean values of ΔFKmax, ΔFKmean, ΔFKsd, ΔFEmax, ΔFEmean, ΔFEsd, ΔCPmax, ΔCPmean, ΔCPsd, ΔBEmax, ΔBEmean, and ΔBEsd between groups were assessed using the Mann-Whitney tests. The diagnostic efficacies of the ΔFKmax, ΔFKmean, ΔFKsd, ΔFEmax, ΔFEmean, ΔFEsd, ΔCPmax, ΔCPmean, ΔCPsd, ΔBEmax, ΔBEmean, and ΔBEsd were assessed using receiver operating characteristic (ROC) curves. The area under the receiver operating characteristic curve (AUROC), sensitivity, and specificity were also calculated. The cut-off *p*-value was set at 0.05.

### Ethics Statement

This study was approved by the Ethics Committee of the Eye and ENT Hospital of Fudan University and was conducted in accordance with the tenets of the Declaration of Helsinki. Signed written informed consents were obtained from all patients.

## Results

**FIGURE 5:**
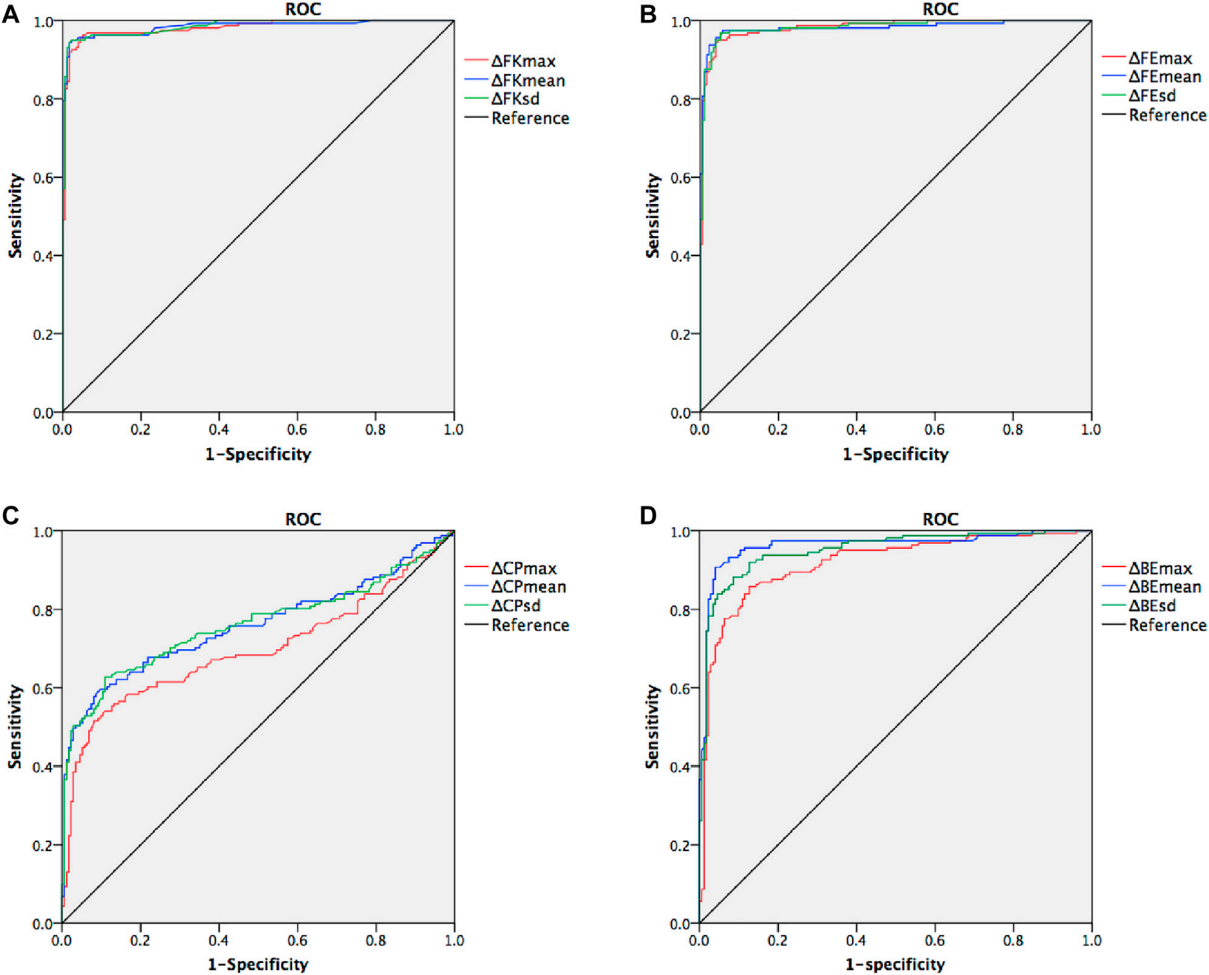
Receiver operating characteristic curves (ROC) for keratoconus versus normal cases. **(A)** combined ROC for ΔFKmax, ΔFKmean, and ΔFKsd **(B)** combined ROC for ΔFEmax, ΔFEmean, and ΔFEsd **(C)** combined ROC for ΔCPmax, ΔCPmean, and ΔCPsd **(D)** combined ROC for ΔBEmax, ΔBEmean, and ΔBEsd.

The mean values for the main anterior segment parameters, including the FK1, FK2, FKm, FKmax, BK1, BK2, BKm, BKmax, TCT, and CTV of the KC and control groups are listed in Table 1. Mann–Whitney tests revealed that the ΔFKmax, ΔFKmean, ΔFKsd, ΔFEmax, ΔFEmean, ΔFEsd, ΔCPmax, ΔCPmean, ΔCPsd, ΔBEmax, ΔBEmean, and ΔBEsd values of the KC group were significantly higher than those of the control group (all *p*-values <0.001) (Table 2). The AUROC values for the ΔFKmax, ΔFKmean, ΔFKsd, ΔFEmax, ΔFEmean, ΔFEsd, ΔCPmax, ΔCPmean, ΔCPsd, ΔBEmax, ΔBEmean, and ΔBEsd for diagnosing keratoconus were widely divergent (Table 3) (Figure 5). However, the ΔFKmean, ΔFKsd, ΔFEsd, and ΔFEmax yielded the four highest accuracies (AUROC = 0.985, 0.985, 0.984, and 0.983, respectively) for discriminating KC cases (including FFK cases) from normal cases (Figures 5A,B). Cut-off values of 0.75 D for the ΔFKmean, 0.67 D for the ΔFKsd, 2.9 μm for the ΔFEsd, and 14.6 μm for the ΔFEmax had the highest sensitivities (95.7, 95.0, 96.9, and 95.0%, respectively) and specificities (96.0, 97.7, 94.8, and 95.4%, respectively). The ΔFKmax, ΔFEmean, ΔBEmean, and ΔBEsd demonstrated acceptable accuracies, with AUROCs of 0.982, 0.982, 0.963, and 0.952, respectively (Figure 5). Cut-off values of 2.56 D for the ΔFKmax, 4.3 μm for the ΔFEmean, 9.9 μm for the ΔBEmean, and 7.6 μm for the ΔBEsd achieved the highest sensitivities (95.7, 95.7, 93.2 and 88.8%) and specificities (95.4, 96.0, 92.5 and 89.1%). The remaining ΔBEmax, ΔCPsd, ΔCPmean, and ΔCPmax values had the lowest accuracies, with AUROCs of 0.918, 0.761, 0.759, and 0.698, respectively (Figures 5C,D). Meanwhile, their sensitivities (85.7, 70.8, 69.6, and 65.2%, respectively) and specificities (85.6, 70.7, 70.7, and 65.5%, respectively) were too low to meet clinical requirements.

**TABLE 1:** Mean values of main topographic parameters.

Parameters	KC group		Control group	
	Mean ± SD	Range	Mean ± SD	Range
FK1 (D)	47.35 ± 6.54	35.82 to 73.38	42.46 ± 1.33	38.41 to 46.09
FK2 (D)	50.88 ± 8.17	37.17 to 83.54	43.46 ± 1.49	38.82 to 47.33
FKm (D)	49.02 ± 7.19	36.48 to 75.04	42.96 ± 1.37	38.66 to 46.66
FKmax (D)	57.64 ± 13.70	40.33 to 117.13	44.27 ± 1.72	39.25 to 54.64
BK1 (D)	−6.85 ± 1.21	−12.20 to −3.64	−5.96 ± 0.21	−6.45 to −5.37
BK2 (D)	−7.60 ± 1.47	−12.98 to −5.60	−6.34 ± 0.25	−7.29 to −5.68
BKm (D)	−7.19 ± 1.29	−12.58 to −5.19	−6.15 ± 0.22	−6.76 to −5.56
BKmax (D)	−9.64 ± 3.28	−24.64 to −5.90	−6.53 ± 0.33	−9.35 to −5.78
CTV (μm)	469.5 ± 53.7	289 to 632	546.6 ± 33.4	464 to 644
TCT (μm)	454.6 ± 57.2	280 to 622	542.1 ± 34.3	381 to 641

SD = standard deviation; KC = keratoconus; FK1 = front flat keratometry; FK2 = front steep keratometry; FKm = front mean keratometry; FKmax = front maximum keratometry; BK1 = back flat keratometry; BK2 = back steep keratometry; BKm = back mean keratometry; BKmax = back maximum keratometry; CTV = corneal thickness at vertex; TCT = corneal thickness at thinnest point; D = dioptor; μm = micron.

**TABLE 2:** Differences in bilateral differential topographic characteristics between groups.

Parameters	KC group	Control group	Z^a^	*p*
	Mean ± SD	Mean ± SD		
ΔFKmax (D)	16.35 ± 13.04	1.50 ± 1.12	−15.254	<0.001
ΔFKmean (D)	6.15 ± 5.05	0.45 ± 0.22	−15.336	<0.001
ΔFKsd (D)	3.94 ± 2.88	0.33 ± 0.21	−15.341	<0.001
ΔFEmax (μm)	44.9 ± 26.9	9.1 ± 4.2	−15.285	<0.001
ΔFEmean (μm)	16.8 ± 11.4	2.4 ± 1.1	−15.254	<0.001
ΔFEsd (μm)	10.8 ± 6.7	1.9 ± 0.9	−15.301	<0.001
ΔBEmax (μm)	81.2 ± 52.8	26.5 ± 18.7	−13.213	<0.001
ΔBEmean (μm)	30.4 ± 22.1	5.8 ± 3.6	−14.648	<0.001
ΔBEsd (μm)	19.4 ± 13.5	4.9 ± 2.7	−14.286	<0.001
ΔCPmax (μm)	58.6 ± 40.2	35.7 ± 14.6	−6.261	<0.001
ΔCPmean (μm)	21.5 ± 13.6	11.8 ± 4.2	−8.204	<0.001
ΔCPsd (μm)	14.7 ± 10.3	7.6 ± 2.7	−8.240	<0.001

ΔFKmax = the maximal front keratometry value within 1.5 mm radius of the bilateral differential topography; ΔFKmean = the mean front keratometry value within 1.5 mm radius of the bilateral differential topography; ΔFKsd = the standard deviation of the front keratometry values within 1.5 mm radius of the bilateral differential topography; ΔFEmax = the maximal front elevation value within 1.5 mm radius of the bilateral differential topography; ΔFEmean = the mean front elevation value within 1.5 mm radius of the bilateral differential topography; ΔFEsd = the standard deviation of the front elevation values within 1.5 mm radius of the bilateral differential topography; ΔBEmax = the maximal back elevation value within 1.5 mm radius of the bilateral differential topography; ΔBEmean = the mean back elevation value within 1.5 mm radius of the bilateral differential topography; ΔBEsd = the standard deviation of the back elevation values within 1.5 mm radius of the bilateral differential topography; ΔCPmax = the maximal corneal pachymetry value within 1.5 mm radius of the bilateral differential topography; ΔCPmean = the mean corneal pachymetry value within 1.5 mm radius of the bilateral differential topography; ΔCPsd = the the standard deviation of the corneal pachymetry values within 1.5 mm radius of the bilateral differential topography; KC = keratoconus; D = dioptor; μm = micron.

a Mann-Whitney test.

**TABLE 3:** Diagnostic efficacy of bilateral differential topographic characteristics.

Parameters	AUROC	Sig.	95% CI	Cut-off	Sensitivity (%)	Specificity (%)
ΔFKmax (D)	0.982	<0.001	0.970∼0.995	2.56	95.7	95.4
ΔFKmean (D)	0.985	<0.001	0.973∼0.997	0.75	95.7	96.0
ΔFKsd (D)	0.985	<0.001	0.974∼0.996	0.67	95.0	97.7
ΔFEmax (μm)	0.983	<0.001	0.972∼0.995	14.6	95.0	95.4
ΔFEmean (μm)	0.982	<0.001	0.968∼0.997	4.3	95.7	96.0
ΔFEsd (μm)	0.984	<0.001	0.972∼0.996	2.9	96.9	94.8
ΔBEmax (μm)	0.918	<0.001	0.887∼0.949	36.3	85.7	85.6
ΔBEmean (μm)	0.963	<0.001	0.941∼0.985	9.9	93.2	92.5
ΔBEsd (μm)	0.952	<0.001	0.929∼0.974	7.6	88.8	89.1
ΔCPmax (μm)	0.698	<0.001	0.639∼0.757	38.4	65.2	65.5
ΔCPmean (μm)	0.759	<0.001	0.705∼0.814	13.2	69.6	70.7
ΔCPsd (μm)	0.761	<0.001	0.706∼0.815	8.5	70.8	70.7

ΔFKmax = the maximal front keratometry value within 1.5 mm radius of the bilateral differential topography; ΔFKmean = the mean front keratometry value within 1.5 mm radius of the bilateral differential topography; ΔFKsd = the standard deviation of the front keratometry values within 1.5 mm radius of the bilateral differential topography; ΔFEmax = the maximal front elevation value within 1.5 mm radius of the bilateral differential topography; ΔFEmean = the mean front elevation value within 1.5 mm radius of the bilateral differential topography; ΔFEsd = the standard deviation of the front elevation values within 1.5 mm radius of the bilateral differential topography; ΔBEmax = the maximal back elevation value within 1.5 mm radius of the bilateral differential topography; ΔBEmean = the mean back elevation value within 1.5 mm radius of the bilateral differential topography; ΔBEsd = the standard deviation of the back elevation values within 1.5 mm radius of the bilateral differential topography; ΔCPmax = the maximal corneal pachymetry value within 1.5 mm radius of the bilateral differential topography; ΔCPmean = the mean corneal pachymetry value within 1.5 mm radius of the bilateral differential topography; ΔCPsd = the the standard deviation of the corneal pachymetry values within 1.5 mm radius of the bilateral differential topography; AUROC = area under the receiver operating characteristic curve; Sig = significance; CI = confidence interval; D = dioptor; μm = micron.

## Discussion

The present study demonstrated a novel algorithm based on the raw data of bilateral differential topography with regard to elevation, keratometry, and pachymetry and then evaluated its efficacy in screening keratoconus and corneal ectasia, achieving desirably high sensitivity and specificity. These findings showed that the evaluation of intereye asymmetry in corneal topography offered unique advantages for detection of high-risk corneas prior to corneal refractive surgeries, which might be a crucial replenishment of unilateral diagnostic models.

The main topographic features of keratoconus include an asymmetry in the corneal topographic pattern (Randleman et al., 2008), corneal thinning, and increased front and back elevations, especially when the thinnest point of the cornea coincides with the highest point of the front and back elevations. Belin and Ambrósio originally created the BAD model, as well as corneal biomechanical property-related models, such as the corvis biomechanical index model and the topographic and biomechanical index model, to distinguish early keratoconus from normal corneas (Vinciguerra et al., 2016; Ambrosio et al., 2017), demonstrating outstanding sensitivity and specificity in the test set. However, in larger sample size studies, the accuracy for diagnosing FFK was significantly decreased with these models (Koc et al., 2019). Moreover, the corneal diameter had an influence on the BAD model, which could compromise the diagnostic accuracy in populations with extremely small or large corneas (Ding et al., 2020). Though the BAD model was sensitive for diagnosing early keratoconus with abnormal features using topography, it could not discriminate FFK, which is defined as a cornea with no abnormal findings on slit-lamp examination or corneal topography but located in the fellow eye of a patient with clinical keratoconus (Ueki et al., 2013). Thus, intereye asymmetry in corneal topography should be a key feature for the identification of FFK. The results of the present study demonstrated that bilateral differential characteristic parameters, including the ΔFKmax, ΔFKmean, ΔFKsd, ΔFEmax, ΔFEmean, ΔFEsd, ΔCPmax, ΔCPmean, ΔCPsd, ΔBEmax, ΔBEmean, and ΔBEsd, were all significantly higher in the KC group than the control group, indicating that intereye asymmetry in topography is vital for distinguishing between keratoconus (corneal ectasia) and a normal cornea; this is similar to the results reported by Henriquez et al. and Eppig et al., composed of 98 and 350 subjects with KC respectively, that revealed significant intereye asymmetry of corneal keratometry, pachymetry, and elevation parameters in KC group (Henriquez et al., 2013; Eppig et al., 2018).

Our ROC analysis showed that the diagnostic efficacies of ΔFKmax, ΔFKmean, ΔFKsd, ΔFEmax, ΔFEmean, ΔFEsd, ΔBEmax, ΔBEmean, and ΔBEsd were remarkably higher than those of the ΔCPmax, ΔCPmean, and ΔCPsd, indicating that changes in the corneal keratometry and elevation values may occur considerably earlier and could be more significant than changes in the corneal thickness in keratoconic eyes. Naderan et al. investigated the relationship between intereye asymmetry in corneal topography and the severity of keratoconus in Caucasian ethnicity. Their results showed that the AUROC values for the central corneal thickness and the corneal thickness at the thinnest point were less than 0.90 and 0.80 for discriminating keratoconus and suspected keratoconus from normal eyes. However, the AUROC values for flat, steep, and mean keratometry findings, as well as front and back elevation values, were higher than 0.95, which was similar to our findings (Naderan et al., 2017). We hypothesize that corneal thinning is associated with lamellar breaks, whereas corneal steepening may be related to bends in the corneal collagen and lamella, which likely occurs much earlier than the breaks. Further studies on cytobiology and molecular biology are required to examine this hypothesis in more detail.

In the present study, it is interesting to note that the ΔFKsd and ΔFEsd both demonstrated outstanding accuracies (both AUROCs >0.98) for discriminating KC and FFK cases from normal cases. These parameters have significant advantages over more conventional topographic parameters, such as the front and back elevation values at the thinnest point and the maximum keratometry value (Naderan et al., 2017). We propose that the ΔFKsd and ΔFEsd typify the overall differences in front keratometry and front elevation (within a 1.5 mm radius) values between eyes, which may be more specific than the keratometry and elevation values obtained at one typical location (such as the thinnest point) for detecting abnormal intereye asymmetry in early keratoconus and even early FFK, which only demonstrates a slight intereye asymmetry on topography without keratoconus detected in either eye.

One limitation of the present study was its relatively small sample size, which made it impossible to optimize the algorithm by subclassifying keratoconus cases according to their severity. The second limitation was that the feature importance of different bilateral differential characteristic parameters was not clarified in the present study. Therefore, permutation importance and impurity-based feature importance analysis of these bilateral differential topographic parameters would be beneficial for improving the diagnostic efficacy of the new algorithm. Future investigations with large sample sizes are necessary to validate this novel algorithm and test out its diagnostic efficacy when distinguish clinical keratoconus, subclinical keratoconus, and FFK respectively from normal corneas.

In summary, bilateral differential topographic parameters may be efficient for the early detection of keratoconus and corneal ectasia. Bilateral differential topographic algorithms may complement conventional diagnostic models by improving the sensitivity and specificity of early keratoconus and ectasia screening before corneal refractive surgeries.

## Data Availability

The raw data supporting the conclusion of this article will be made available by the authors, without undue reservation.

## Glossary

AUROC: area under the receiver operating characteristic curve
AS: anterior segment
BAD: Belin/Ambrósio Enhanced Ectasia total deviation index
BK1: back flat keratometry
BK2: back steep keratometry
BKmax: back maximum keratometry
BKm: back mean keratometry
CTV: corneal thickness at vertex
D: diopter
FFK: forme fruste keratoconus
FK1: front flat keratometry
FK2: front steep keratometry
FKmax: front maximum keratometry
FKm: front mean keratometry
KC: keratoconus
OCT: optical coherence tomography
ROC: receiver operating characteristic
SD: standard deviation
Sig: significance
TCT: corneal thickness at thinnest point
ΔBEmax: maximal back elevation value within a 1.5-mm radius of the bilateral differential topography
ΔBEmean: mean back elevation value within a 1.5-mm radius of the bilateral differential topography
ΔBEsd: standard deviation of the back elevation values within a 1.5-mm radius of the bilateral differential topography
ΔCPmax: maximal corneal pachymetry value within a 1.5-mm radius of the bilateral differential topography
ΔCPmean: mean corneal pachymetry value within a 1.5-mm radius of the bilateral differential topography
ΔCPsd: standard deviation of the corneal pachymetry values within a 1.5-mm radius of the bilateral differential topography
ΔFEmax: maximal front elevation value within a 1.5-mm radius of the bilateral differential topography
ΔFEmean: mean front elevation value within a 1.5-mm radius of the bilateral differential topography
ΔFEsd: standard deviation of the front elevation values within a 1.5-mm radius of the bilateral differential topography
ΔFKmax: maximal front keratometry value within a 1.5-mm radius of the bilateral differential topography
ΔFKmean: mean front keratometry value within a 1.5-mm radius of the bilateral differential topography
ΔFKsd: standard deviation of the front keratometry values within a 1.5-mm radius of the bilateral differential topography
μm: micron
